# Association between sarcoidosis and HLA polymorphisms in a Czech population from Central Europe: focus on a relationship with clinical outcome and treatment

**DOI:** 10.3389/fmed.2023.1094843

**Published:** 2023-04-21

**Authors:** K. Sikorova, K. Osoegawa, L. Kocourkova, A. Strnad, J. Petrkova, M. A. Fernández-Viña, M. Doubkova, M. Petrek

**Affiliations:** ^1^Department of Pathological Physiology, Faculty of Medicine and Dentistry, Palacký University Olomouc, Olomouc, Czechia; ^2^Histocompatibility & Immunogenetics Laboratory, Stanford Blood Center, Palo Alto, CA, United States; ^3^Laboratory of Cardiogenomics–Experimental Medicine, University Hospital Olomouc, Olomouc, Czechia; ^4^Histocompatibility, Immunogenetics, and Disease Profiling Laboratory, Department of Pathology, Stanford Blood Center, Stanford University School Medicine, Palo Alto, CA, United States; ^5^Department of Pulmonary Diseases and Tuberculosis, Faculty of Medicine of Masaryk University, University Hospital Brno, Brno, Czechia

**Keywords:** sarcoidosis, HLA, Czech, clinical course, Löfgren’s syndrome, biomarker, inflammatory disorders

## Abstract

**Background:**

Sarcoidosis is an immune-mediated systemic disease with unknown etiology affecting the lung predominantly. The clinical manifestation of sarcoidosis is rather diverse ranging from Löfgren’s syndrome to fibrotic disease. Also, it differs among patients with distinct geographical and ethnic origins, consistent with environmental and genetic factors’ role in its pathogenesis. Of those, the polymorphic genes of the HLA system have been previously implicated in sarcoidosis. Therefore, we have performed an association study in a well-defined cohort of Czech patients aiming to define how variation in HLA genes, may contribute to disease origin and development.

**Materials and methods:**

Total of the 301 Czech unrelated sarcoidosis patients were diagnosed according to international guidelines. In those, HLA typing was performed using next-generation sequencing. The allele frequencies at six HLA loci (*HLA-A,-B,-C,-DRB1,-DQA1*, and -*DQB1*) observed in the patients were compared with HLA allele distribution determined in 309 unrelated healthy Czech subjects; sub-analyses of relationships between HLA and distinct sarcoidosis clinical phenotypes were performed. Associations were assessed by two-tailed Fischer’s exact test with correction for multiple comparisons.

**Results:**

We report two variants, HLA-DQB1*06:02, and HLA-DQB1*06:04, as risk factors for sarcoidosis, and three variants, HLA-DRB1*01:01, HLA-DQA1*03:01, and HLA-DQB1*03:02 as protective factors. HLA-B*08:01, HLA-C*07:01, HLA-DRB1*03:01, HLA-DQA1*05:01, and HLA-DQB1*02:01 variants associated with Löfgren’s syndrome, a more benign phenotype. HLA- DRB1*03:01 and HLA-DQA1*05:01 alleles were connected with better prognosis—chest X-ray (CXR) stage 1, disease remission, and non-requirement of corticosteroid treatment. The alleles HLA-DRB1*11:01 and HLA-DQA1*05:05 are associated with more advanced disease represented by the CXR stages 2−4. HLA-DQB1*05:03 associated with sarcoidosis extrapulmonary manifestation.

**Conclusion:**

In our Czech cohort, we document some associations between sarcoidosis and HLA previously described in other populations. Further, we suggest novel susceptibility factors for sarcoidosis, such as HLA-DQB1*06:04, and characterize associations between HLA and sarcoidosis clinical phenotypes in Czech patients. Our study also extends the role of the 8.1 ancestral haplotype (HLA-A*01:01∼HLA-B*08:01∼HLA-C*07:01∼HLA-DRB1*03:01∼HLA-DQA1*05:01∼HLA-DQB1*02:01), already implicated in autoimmune diseases, as a possible predictor of better prognosis in sarcoidosis. The general translational application of our newly reported findings for personalized patient care should be validated by an independent study from another, international referral center.

## 1. Introduction

Sarcoidosis is a multi-systemic inflammatory disease characterized by non-caseating granulomas of unknown etiology that majorly affect the lung but can affect any organ system. The presentation of sarcoidosis is diverse, and the course of the disease can vary from acute to sub-acute inflammatory Löfgren’s syndrome (LS) to progressive form, which occurs in almost 20% of patients (1) and may eventually include fibrosis.

The prevalence and incidence of sarcoidosis vary substantially by ethnicity, region, sex, and age of onset. The lowest incidence and prevalence are in the Asians; in the Caucasian and African American populations, the incidence is more pronounced. The incidence of sarcoidosis in the Czech population is 3.1 per 100,000 inhabitants; its prevalence is 63.1 per 100,000 (2, 3). In Czech patients, apart from the lungs, another extrapulmonary manifestation has also been reported; extrapulmonary impairment is present in 10–90% of patients, depending on the analysis (4, 5).

The genetic background of individuals has been suggested as an essential factor in sarcoidosis susceptibility. Several genetic association studies have supported the genetic basis of sarcoidosis, its sub-phenotypes, and clinical course (6, 7). Studies also reported different genetic variants based explicitly on the ethnic background of the studied subjects (8). The crucial role of the immunogenetic polymorphic system, HLA (Human Leukocyte Antigen), in susceptibility to sarcoidosis has been shown by some previous investigators enrolling mostly patients of North European descent (7, 9, 10); a previous study in a group of 114 Czech, i.e., Central European patients, was limited to a single class II (*HLA-DRB1*) locus (11).

To investigate a possible role of HLA polymorphisms across class I and II loci in sarcoidosis in this particular population, we enrolled three hundred one well-defined Czech patients and employed precise NGS (Next Generation Sequencing) genotyping focusing on associations between HLA and sarcoidosis clinical phenotypes.

## 2. Patients and methods

### 2.1. Patients and control subjects

A total of 301 sarcoidosis patients enrolled in this study were diagnosed and followed according to the ATS (American Thoracic Society), ERS (European Respiratory Society), and WASOG (World Association of Sarcoidosis and other Granulomatous Disorders) statement on sarcoidosis (12) at the Department of Pneumology and Phtiseology, University Hospital and Faculty of Medicine, Brno, the Czech Republic. The control group consisted of 309 unrelated healthy blood donors from the same geographical region of the Czech Republic, the place of sampling was Olomouc, a city located close (70 km) to Brno. Both patients and control subjects were of Czech ethnicity as assessed by place of birth, surname, and Czech as a native language. For the characteristics of patients and control subjects see Table 1. Study participants provided informed consent with participation in the study, which was approved by the institutional review board–the Ethic committee of the University Hospital and the Faculty Medicine Palacký University in Olomouc, vote on project NV18-05-00134 dated 27.06.2017).

**TABLE 1:** Characteristics of the patients and healthy control subjects enrolled in the study.

	Patients	Controls
*N*	301	309
Age (mean ± SD), years	46.77 ± 11.49	32.38 ± 8.63
Age (range), years	24–72	18–61
M/F	149/152	143/166
CXR I	95	−
CXR II	142	−
CXR III	58	−
CXR IV	6	−
Löfgren’s syndrome: yes/no	55/244	−
Extrapulmonary manifestation: yes/no	91/210	−
Steroid treatment: yes/no	194/105	−
Disease course: remission/progression	142/134	−

Data on the age of patients are provided as mean ± standard error of the mean; age range data as the minimum and maximum. N, number of subjects; SD, standard deviation; M, male; F, female; CXR, chest X-ray stage. The patients were followed for at least 2 years after the diagnosis in order to observe the development of the disease (information on disease course was not available for 25 patients) and patients response to treatment (treatment information was not available for 2 patients). Löfgren’s syndrome data was not available for two patients. Concerning the extrapulmonary manifestation of sarcoidosis, generalized lymphadenopathy was observed in 25 patients (localized lymph node involvement in 16 patients), skin involvement in 25, spleen involvement in 16 patients and thrombocytopenia in 2 patients. Other extrapulmonary locations were: liver (*n* = 8), heart (*n* = 8), eyes (*n* = 4), nervous system (*n* = 4), bones (*n* = 3), marrow (*n* = 1), soft tissue (*n* = 1), epididymis (*n* = 1), parotid gland (*n* = 1), salivary gland (*n* = 1), tonsils (*n* = 1), and intestines (*n* = 1).

Genomic DNA from peripheral blood samples obtained from patients and control subjects was isolated using the Arrow automated extraction system (Isogen Life Science, PW De Meern, Utrecht, Netherlands).

### 2.2. NGS HLA analysis

To determine HLA alleles, DNA samples obtained from the study subjects were sequenced using the Omixon Holotype HLA 96/11 and 96/7 (Omixon Biocomputing Ltd., Budapest, Hungary) kit on the Illumina MiSeq next-generation sequencing platform (Illumina, San Diego, CA, USA). Long-range PCR amplified the HLA class I and class II loci. All seven amplicons from each sample were pooled into a final 35 μl volume on a new 96-well PCR plate and purified from residual primers and unincorporated nucleotides. Then follows library preparation with the fragmentation of pooled amplicons, fragment end repair, and ligation of sample-specific indexed adaptors. Equal aliquots of indexed sample-specific libraries were combined into one pooled library to carry out magnetic bead-based library clean-up. The concentration of the size selected library was determined on LightCycler 480 II (Roche Diagnostics, Mannheim, Germany) real-time PCR instrument using KAPA Sybr Fast qPCR Master Mix (KAPA Biosystems, Boston, MA, USA). Before sequencing, the library was denatured by NaOH, diluted with hybridization buffer, and a 9 pM library was loaded on a MiSeq flow cell (Illumina, San Diego, CA, USA) sequenced in a single 300 cycle (V2) paired-end sequencing run. Collected reads were exported in fastq format (13).

Allele assignment was performed with the Omixon Twin software v4.1.0.–v4.4.1. and the IPD-IMGT/HLA database Release 3.34−3.45.

The success rate of NGS HLA-typing in all seven loci is presented in Supplementary Table 1.

The *HLA-DPB1* locus was excluded from further analyses due to a suboptimal genotyping score in the patients (84%). *HLA-DQB1* locus was genotyped in all control samples and in 205 out of 301 patient samples.

### 2.3. Statistical analysis

Frequencies of the determined HLA alleles were obtained by direct counting (Supplementary Tables 2, 3). Association between the allele frequency and sarcoidosis and its clinical phenotypes was evaluated by two-sided Fisher’s exact probability test, providing odds ratio (OR), 95% confidence interval (CI), and level of significance (*p*-value < 0.05). Bonferroni’s correction for multiple comparisons was performed for each HLA loci as *p*_*corr*_ = 1-(1-*p*)*^n^* (*n* of variants in loci at four digits). The statistical analysis was performed using^1^ (9.09.2022), 2 × 2 Contingency Table vassarstats.net (9.09.2022) and excel tables; *p* < 0.05. For subanalyses between clinical phenotypes, an additional parameter-carriage rate-was employed; this parameter, also known as HLA “antigen frequency,” was determined as a number of individuals carrying a particular variant divided by the number of all individuals tested for the given locus; comparisons using this data are shown in Supplementary Tables 4−7.

## 3. Results

### 3.1. Comparison of the distribution of HLA variants between patients and healthy population

We analyzed 6 HLA loci (*HLA-A,-B,-C,-DRB1,-DQA1*, and *-DQB1*) in 301 unrelated patients with sarcoidosis diagnosed and followed according to the ATS, ERS, and WASOG statement on sarcoidosis (12) at the Department of Pneumology and Phtiseology, University Hospital and Faculty of Medicine, Brno, Czechia and 309 unrelated Czech healthy control subjects, blood donors from the same geographical region of Czechia. The numbers of the determined variants in the typed loci in patients and control subjects are shown in Supplementary Table 1 and frequencies of determined variants in Supplementary Tables 2, 3.

When comparing the distribution of HLA polymorphisms between sarcoidosis patients and control subjects, we observed 18 HLA variants associated with sarcoidosis on the primary level, 3 of those in HLA class I and 15 in HLA class II, respectively; for numbers that correspond to loci see Supplementary Table 1 (the right column).

Table 2 shows the variants which remained associated with the disease after the correction for multiple comparisons. All were within HLA class II loci. HLA-DQB1*06:02 and HLA-DQB1*06:04 can be considered as susceptibility (risk) factors for sarcoidosis; the frequency of the HLA-DQB1*06:02 in the patients was increased 1.75 times compared with the control subjects, and the HLA-DQB1*06:04 frequency increased four times in the patients. HLA-DRB1*01:01, HLA-DQA1*03:01, and HLA-DQB1*03:02 may confer protection against sarcoidosis: frequency of HLA-DRB1*01:01 decreased in the patients 2.5 times compared with the healthy control subjects, HLA-DQA1*03:01 dropped two times, and HLA-DQB1*03:02 4.5 times.

**TABLE 2:** Summary of the observed associations between HLA alleles and sarcoidosis in the investigated Czech patients (only the alleles associated at least on primary level are shown).

HLA	*f* patiens *n* = 301	*f* controls *n* = 309	OR (95% CI)	*p*-value	*p*_*corr*_
HLA-A*26:01	0.027	0.051	0.512 (0.276−0.95)	0.035	0.632
HLA-B *07:02	0.149	0.101	1.553 (1.097−2.199)	0.014	0.494
HLA-B*08:01	0.149	0.104	1.499 (1.062−2.115)	0.024	0.679
HLA-DRB1*01:01	0.027	0.068	**0.378 (0.21−0.682)**	**0.001**	**0.041**
HLA-DRB1*04:01	0.037	0.063	0.57 (0.333−0.976)	0.046	0.87
HLA-DRB1*07:01	0.093	0.148	0.591 (0.415−0.843)	0.004	0.142
HLA-DRB1*11:01	0.11	0.071	1.621 (1.085−2.423)	0.021	0.592
HLA-DRB1*13:02	0.048	0.02	2.518 (1.273−4.983)	0.007	0.249
HLA-DRB1*14:54	0.033	0.015	2.291 (1.035−5.074)	0.039	0.822
HLA-DRB1*15:01	0.188	0.132	1.529 (1.119−2.088)	0.008	0.28
HLA-DQA1*01:01	0.035	0.073	0.461 (0.27−0.787)	0.004	0.073
HLA-DQA1*01:02	0.272	0.201	1.48 (1.129−1.941)	0.005	0.082
HLA-DQA1*02:01	0.094	0.148	0.595 (0.416−0.85)	0.004	0.07
HLA-DQA1*03:01	0.042	0.087	**0.459 (0.281−0.752)**	**0.002**	**0.032**
HLA-DQB1*03:02	0.021	0.096	**0.198 (0.094−0.419)**	**8.49 × 10^–07^**	**2.38 × 10^–05^**
HLA-DQB1*05:01	0.052	0.088	0.564 (0.332−0.957)	0.035	0.63
HLA-DQB1*06:02	0.209	0.119	**1.955 (1.384−2.763)**	**1.53 × 10^–04^**	**0.004**
HLA-DQB1*06:04	0.054	0.013	**4.334 (1.9−9.887)**	**3.02 × 10^–04^**	**0.008**

OR, odds ratio; CI, confidence interval; *p_corr_–p*-value after the correction for multiple comparisons (significant values highlighted in bold), NA, not analyzed.

### 3.2. HLA variants in patients with Löfgren’s syndrome

We performed several subgroup analyses to evaluate the relationship between HLA and sarcoidosis clinical phenotypes. We first compared the patients with Löfgren’s syndrome (LS), a benign sarcoidosis phenotype, and those with non-Löfgren sarcoidosis. Ten variants were associated with LS on the primary level, and six remained significantly associated after correction for multiple comparisons (Table 3). Regarding HLA class I, variants HLA-A*01:01 (*p*_*corr*_ = 3.86 × 10^–04^), HLA-B*08:01 (*p*_*corr*_ = 2.50 × 10^–04^), and HLA-C*07:01 (*p*_*corr*_ = 2.46 × 10^–04^) were overrepresented in the patients with LS (Table 3). When comparing carriage rates in LS and non-LS groups, HLA-B*08:01 (*p*_*corr*_ = 9.34 × 10^–05^) and HLA-C*07:01 (*p*_*corr*_ = 0.002) again appeared more frequently (Supplementary Table 4), and can be considered risk factors for LS.

**TABLE 3:** Comparison of the HLA allele frequencies between the patients with Löfgren’s syndrome, LS (*n* = 55) compared with the patients with non-LS (*n* = 244).

HLA	*f* LS *n* = 55	*f* non-LS *n* = 244	*f* controls *n* = 309	OR (95% CI)	*p*-value	*p*_*corr*_
HLA-A*01:01	**0.336**	**0.146**	**0.153**	**2.977 (1.863−4.757)**	**1.38 × 10^–05^**	**3.86 × 10^–04^**
HLA-B*08:01	**0.3**	**0.116**	**0.104**	**3.283 (2.004−5.38)**	**5.33 × 10^–06^**	**2.50 × 10^–04^**
HLA-B*41:02	0.027	0	0.01	NA	6.18 **×** 10**^–^**^03^	0.253
HLA-C*03:03	0.009	0.055	0.041	0.157 (0.021−1.165)	0.043	0.729
HLA-C*04:01	0.036	0.121	0.121	0.274 (0.097−0.772)	8.93 **×** 10^–03^	0.236
HLA-C*07:01	**0.355**	**0.158**	**0.15**	**2.932 (1.85**−**4.646)**	**8.20 × 10^–06^**	**2.46 × 10^–04^**
HLA-C*07:18	0.018	0	0.003	NA	0.034	0.641
HLA-C*17:03	0.027	0	0.011	NA	6.09 **×** 10^–03^	0.167
HLA-DRB1*03:01	**0.318**	**0.082**	**0.11**	**5.203 (3.108**−**8.712)**	**1.20 × 10^–09^**	**5.16 × 10^–08^**
HLA-DRB1*11:01	0.046	0.124	0.071	0.338 (0.132−0.863)	0.017	0.522
HLA-DQA1*05:01	**0.318**	**0.075**	**0.106**	**5.781 (3.418**−**9.78)**	**2.01 × 10^–10^**	**3.42 × 10^–09^**
HLA-DQB1*02:01	**0.267**	**0.074**	**0.109**	**4.576 (2.256**−**9.281)**	**5.98 × 10^–05^**	**1.67 × 10^–03^**
HLA-DQB1*03:04	0.033	0	0.007	NA	0.024	0.491
HLA-DQB1*05:03	0	0.068	0.031	NA	0.033	0.611

For the legend see Table 2.

Regarding HLA class II, 3 variants associated with LS: HLA-DRB1*03:01 (*p*_*corr*_ = 5.16 × 10^–08^); HLA-DQA1*05:01 (*p*_*corr*_ = 3.42 × 10^–09^) and HLA-DQB1*02:01 (*p*_*corr*_ = 1.67 × 10^–03^) (Table 3). Two of these variants were associated with LS when carriage rates were used for comparisons–HLA-DRB1*03:01 (*p*_*corr*_ = 1.93 × 10^–08^) and HLA-DQA1*05:01 (*p*_*corr*_ = 0.02) (Supplementary Table 4).

### 3.3. Comparison of the distribution of HLA variants in the context of different chest radiography stages

Next, we were interested if there is an association between the distinct CXR stage(s) of sarcoidosis and particular HLA variants. Therefore, the observed HLA frequencies were compared between the patient subgroups separated according to the CXR stages. The patients were divided into two groups—in the first group there were patients with CXR stage 1 sarcoidosis, and in the second group, patients with sarcoidosis CXR stage 2−4. We observed nine variants associated with CXR stage 1 on the primary level; two variants remained associated after correction for multiple comparisons (Table 4). Both these variants (HLA-DRB1*03:01 and HLA-DQA1*05:01) occurred three times more often in the CXR 1 patients than in patients with higher CXR stages. In comparisons based on carriage rates, apart from HLA-DRB1*03:01 (*p*_*corr*_ = 6.29 × 10^–05^) and HLA-DQA1*05:01 (*p*_*corr*_ = 3.63 × 10^–06^) CXR stage 1 associated also with HLA-B*08:01 (*p*_*corr*_ = 0.042) and HLA-C*07:01 (*p*_*corr*_ = 0.011) (Supplementary Table 5).

**TABLE 4:** Comparison of the HLA allele frequencies between the patients with chest X-ray (CXR) stage 1 (*n* = 95) compared with the patients with stages 2–4 (*n* = 206); only the alleles associated at least on the primary level are shown.

HLA	*f* stg. 1 *n* = 95	*f* stg. 2, 3, 4 *n* = 206	*f* controls *n* = 309	OR (95% CI)	*p*-value	*p*_*corr*_
HLA-A*01:01	0.247	0.148	0.153	1.891 (1.234−2.899)	4.17 **×** 10^–03^	0.110
HLA-B*08:01	0.221	0.115	0.104	2.186 (1.383−3.455)	1.21 **×** 10^–03^	0.055
HLA-B*35:01	0.011	0.047	0.05	0.218 (0.05−0.947)	0.030	0.758
HLA-B*38:01	0.074	0.032	0.035	2.423 (1.116−5.262)	0.032	0.786
HLA-B*41:02	0.016	0.000	0.01	NA	0.032	0.779
HLA-B*44:03	0.011	0.042	0.033	0.245 (0.056−1.073)	0.046	0.892
HLA-C*07:01	0.258	0.163	0.15	1.789 (1.179−2.716)	7.50 **×** 10^–03^	0.202
HLA-C*17:03	0.016	0.000	0.011	NA	0.031	0.612
HLA-DRB1*03:01	**0.226**	**0.078**	**0.11**	**3.455 (2.105−5.672)**	**1.02 × 10^–06^**	**4.39 × 10^–05^**
HLA-DRB1*11:01	**0.037**	**0.144**	**0.071**	**0.228 (0.102−0.508)**	**3.70 × 10^–05^**	**1.59 × 10^–03^**
HLA-DQA1*05:01	**0.220**	**0.073**	**0.106**	**3.582 (2.154−5.954)**	**9.52 × 10^–07^**	**1.62 × 10^–05^**
HLA-DQA1*05:05	**0.134**	**0.251**	**0.189**	**0.463 (0.287−0.746)**	**1.19 × 10^–03^**	**0.020**
HLA-DQB1*02:01	0.181	0.078	0.109	2.601 (1.323−5.115)	0.010	0.245

For the legend see Table 2.

Of the four variants associated with higher CXR stages on the primary level, two (HLA-DRB1*11:01, HLA-DQA1*05:05) remained associated after correction for multiple comparisons (Table 4). HLA-DRB1*11:01 frequency was four times higher in CXR stages 2−4 than in CXR stage 1 and this variant was associated with higher sarcoidosis CXR stages (*p*_*corr*_ = 0.001) also when comparing carriage rates (Supplementary Table 5).

### 3.4. HLA variants in the context of clinical disease course

We were also interested if HLA variation can be linked to the clinical course of the disease. We, therefore, compared the patients undergoing remission and the patients in whom the disease persisted; the disease status was assessed 2 years after the diagnosis. Eight variants were associated with remission on the primary level, two variants remain associated after correction for multiple comparisons (Table 5). Patients with variants HLA-DRB1*03:01 or HLA-DQA1*05:01 were three times more likely to develop remission of the disease. Those two variants were significantly associated with remission also in comparison of carriage rates, HLA-DRB1*03:01 (*p*_*corr*_ = 1.65 × 10^–04^) and HLA-DQA1*05:01 (*p*_*corr*_ = 6.03 × 10^–05^) (Supplementary Table 6). We observed eight variants associated with persistent sarcoidosis on the primary level of significance (Table 5).

**TABLE 5:** Comparison of the HLA allele frequencies between the patients with disease remission (*n* = 142) compared with the patients with persistent disease course (*n* = 134); only the alleles associated at least on the primary level are shown.

HLA	*f* rem. *n* = 142	*f* pers. *n* = 134	*f* controls *n* = 309	OR (95% CI)	*p*-value	*p*_*corr*_
HLA-A*01:01	0.236	0.138	0.153	1.928 (1.239−3)	3.33 **×** 10^–03^	0.089
HLA-B*08:01	0.191	0.105	0.104	2.013 (1.232−3.289)	5.68 **×** 10^–03^	0.235
HLA-B*35:03	0.050	0.011	0.025	4.58 (1.301−16.122)	0.012	0.433
HLA-B*38:01	0.067	0.030	0.035	2.33 (1.002−5.417)	0.049	0.907
HLA-B*44:03	0.014	0.052	0.033	0.259 (0.084−0.797)	0.015	0.509
HLA-DRB1*03:01	**0.190**	**0.060**	**0.11**	**3.668 (2.042−6.591)**	**4.98 × 10^–06^**	**2.14 × 10^–04^**
HLA-DRB1*07:01	0.060	0.124	0.148	0.45 (0.244−0.828)	0.011	0.384
HLA-DRB1*09:01	0.000	0.019	0.012	NA	0.026	0.676
HLA-DRB1*11:01	0.070	0.150	0.071	0.428 (0.243−0.753)	3.76 **×** 10^–03^	0.150
HLA-DRB1*14:54	0.014	0.056	0.015	0.239 (0.078−0.73)	8.74 **×** 10^–03^	0.314
HLA-DQA1*01:04	0.018	0.064	0.029	0.266 (0.097−0.732)	7.90 **×** 10^–03^	0.126
HLA-DQA1*02:01	0.061	0.124	0.148	0.456 (0.248−0.841)	0.012	0.179
HLA-DQA1*05:01	**0.182**	**0.060**	**0.106**	**3.48 (1.93−6.275)**	**1.24 × 10^–05^**	**2.11 × 10^–04^**
HLA-DQB1*05:03	0.020	0.085	0.031	0.219 (0.063−0.768)	0.015	0.351
HLA-DQB1*06:01	0.027	0.000	0.016	NA	0.038	0.661
HLA-DQB1*06:09	0.027	0.000	0.005	NA	0.038	0.661

For the legend see Table 2.

### 3.5. HLA variants in the context of corticosteroid treatment

Treatment requirement may be considered as another indicator of sarcoidosis disease course. Our further subanalysis examined whether corticosteroid treatment is connected to a particular HLA variant. We compared the patients who received corticosteroid treatment with those who did not receive it. Nine HLA variants were primarily associated with no need of treatment; three variants (HLA-A*01:01, HLA-DRB1*03:01, and HLA-DQA1*05:01) remained associated after correction for multiple comparisons (Table 6), they occurred twice more often in non-treated patients. The same three variants were associated with non-treated patients also in comparison of carriage rates: HLA-A*01:01 (*p*_*corr*_ = 1.45 × 10^–04^), HLA-DRB1*03:01 (*p*_*corr*_ = 0.024), and HLA-DQA1*05:01 (*p*_*corr*_ = 0.007) (Supplementary Table 7). By contrast, one variant associated with the need for treatment: HLA-A*02:01 (*p*_*corr*_ = 0.038) (Supplementary Table 7).

**TABLE 6:** Comparison of the HLA allele frequencies between the patients with treatment (*n* = 194) compared with the patients without treatment (*n* = 105); only the alleles associated at least on the primary level are shown.

HLA	*f* treatment *n* = 194	*f* no treatment *n* = 105	*f* controls *n* = 309	OR (95% CI)	*p*-value	*p*_*corr*_
HLA-A*01:01	**0.137**	**0.262**	0.153	**0.446 (0.292−0.68)**	**2.18 × 10^–04^**	**6.09 × 10^–03^**
HLA-A*33:03	0.000	0.014	0.003	NA	0.043	0.707
HLA-B*08:01	0.127	0.192	0.104	0.609 (0.386−0.961)	0.040	0.854
HLA-B*35:01	0.021	0.058	0.05	0.345 (0.139−0.857)	0.029	0.748
HLA-B*58:01	0.000	0.014	0.005	NA	0.042	0.869
HLA-C*04:01	0.085	0.143	0.121	0.558 (0.33−0.944)	0.036	0.665
HLA-DRB1*03:01	**0.088**	**0.195**	**0.11**	**0.398 (0.244−0.65)**	**2.60 × 10^–04^**	**0.011**
HLA-DRB1*11:01	0.135	0.067	0.071	2.18 (1.177−4.035)	0.013	0.440
HLA-DQA1*05:01	**0.085**	**0.186**	**0.106**	**0.406 (0.246−0.67)**	**4.85 × 10^–04^**	**8.21 × 10^–03^**
HLA-DQA1*05:05	0.242	0.162	0.189	1.657 (1.068−2.57)	0.027	0.370
HLA-DQB1*02:01	0.076	0.176	0.109	0.383 (0.197−0.745)	5.25 × 10^–03^	0.137
HLA-DQB1*05:03	0.076	0.009	0.031	8.743 (1.161−65.827)	0.012	0.289

For the legend see Table 2.

### 3.6. HLA variants in patients with an extrapulmonary manifestation of sarcoidosis

Our final subanalysis focused on a possible link between HLA and the extrapulmonary manifestation of sarcoidosis, which was reported in 91 (30%) of our patients. When comparing patients with the extrapulmonary manifestation of sarcoidosis with the patients without it, we observed one variant associated with extrapulmonary manifestation–HLA-DQB1*05:03 (*p* = 0.033; OR; 95% CI = [2.653; 1.114−6.318]); its frequency in patients with extrapulmonary manifestation was doubled. By contrast, HLA-DRB1*01:01 (*p* = 0.049; OR; 95% CI = [0.151; 0.02−1.151]) occurred six times more often in patients without extrapulmonary manifestation; both these associations were detected on the primary level.

### 3.7. Linkage disequilibrium between associated HLA variants

Linkage disequilibrium between the alleles associated with sarcoidosis in Czech patients was calculated. A non-random association was identified between variants associated with the risk of sarcoidosis. The strongest linkage was detected between HLA-DQB1*06:02-HLA-DRB1*15:01. In the context of variants protective from sarcoidosis, the strong association was (among others) between alleles HLA-DQA1*03:01-HLA-DQB1*03:02. Regarding variants associated with higher stages of the disease there was a linkage disequilibrium between variants HLA-DQA1*05:05-HLA-DRB1*11:01. A strong linkage disequilibrium was detected between variants HLA-DQA1*05:01-HLA-DRB1*03:01 which related both to CXR stage 1 and to disease remission. There was also a linkage between variants HLA-A*01:01-HLA-DRB1*03:01 associated with no need for treatment. The variants associated with LS, i.e., HLA-A*01:01, HLA-B*08:01, HLA-C*07:01, HLA-DRB1*03:01, HLA-DQA1*05:01, and HLA-DQB1*02:01 were found in linkage with each other except for HLA-A*01:01- HLA-DQB1*02:01 (Table 7).

**TABLE 7:** Linkage disequilibrium between HLA alleles observed to be associated with sarcoidosis in Czech population among variants being susceptible for sarcoidosis, protective for sarcoidosis, associated with unfavorable prognosis and associated with better prognosis in sarcoidosis.

HLA1	HLA2	*p*-value
**Risk**		
HLA-DQB1*06:02	HLA-DRB1*15:01	1.18 × 10^–36^
HLA-DQA1*01:02	HLA-DQB1*06:02	9.41 × 10^–27^
HLA-DQB1*06:04	HLA-DRB1*13:02	3.70 × 10^–19^
HLA-B*07:02	HLA-DQB1*06:02	2.95 × 10^–07^
**Protective**		
HLA-DQA1*01:01	HLA-DRB1*01:01	1.05 × 10^–19^
HLA-DQA1*03:01	HLA-DRB1*04:01	3.74 × 10^–11^
HLA-DQA1*03:01	HLA-DQB1*03:02	8.79 × 10^–08^
HLA-DQB1*05:01	HLA-DRB1*01:01	4.86 × 10^–07^
**Unfavorable prognosis**		
HLA-DQA1*05:05	HLA-DRB1*11:01	8.54 × 10^–24^
HLA-DQA1*01:04	HLA-DQB1*05:03	6.25 × 10^–18^
HLA-DQA1*01:04	HLA-DRB1*14:54	6.09 × 10^–24^
HLA-DQB1*05:03	HLA-DRB1*14:54	2.23 × 10^–13^
**Better prognosis**		
HLA-DQA1*05:01	HLA-DRB1*03:01	6.36 × 10^–59^
HLA-B*08:01	HLA-C*07:01	1.29 × 10^–49^
HLA-B*08:01	HLA-DRB1*03:01	8.08 × 10^–32^
HLA-B*08:01	HLA-DQA1*05:01	9.74 × 10^–32^
HLA-C*07:01	HLA-DQA1*05:01	9.25 × 10^–23^
HLA-C*07:01	HLA-DRB1*03:01	9.90 × 10^–23^
HLA-A*01:01	HLA-B*08:01	2.02 × 10^–22^
HLA-DQA1*05:01	HLA-DQB1*02:01	3.62 × 10^–22^
HLA-DQB1*02:01	HLA-DRB1*03:01	1.17 × 10^–21^
HLA-A*01:01	HLA-C*07:01	5.83 × 10^–20^
HLA-A*01:01	HLA-DRB1*03:01	7.73 × 10^–15^
HLA-A*01:01	HLA-DQA1*05:01	9.73 × 10^–15^
HLA-B*08:01	HLA-DQB1*02:01	2.14 × 10^–13^
HLA-C*07:01	HLA-DQB1* 02:01	5.01 × 10^–10^

Number of distinct two-way tests performed = N2 = 23706, *p*-value for two-way comparisons = 0.05/N2 = 2.109 × 10^–06^ for a 95% confidence level.

## 4. Discussion

This study, performed in the Czech population, investigated the association of HLA polymorphisms with sarcoidosis and focused on associations between HLA and sarcoidosis clinical phenotypes. Observations of an association between sarcoidosis and HLA-DQB1*06:04 as a susceptibility and of HLA-DQA1*03:01 as a protective marker in the Czech population represent novel findings. Further, this study, including altogether 610 subjects, confirmed associations between HLA-DQB1*06:02 (susceptibility) and HLA-DRB1*01:01, HLA-DQB1*03:02 (protection), previously reported in smaller-scale studies in other, namely North-European and also East-Asian populations (13–17). Regarding clinical phenotypes of sarcoidosis, HLA-A*01:01, HLA-B*08:01, HLA-C*07:01, HLA-DRB1*03:01, HLA-DQA1*05:01, and HLA-DQB1*02:01 associated with Löfgren’s syndrome. The variants HLA-DRB1*03:01 and HLA-DQA1*05:01 were associated with disease remission and the presence of CXR stage 1, while the variants HLA-DRB1*11:01 and HLA-DQA1*05:05 associated with CXR stages 2−4. Associations were also observed with other clinically relevant disease features, i.e., non-requirement of corticosteroid treatment and systemic disease: i.e., HLA-A*02:01 was overrepresented in patients needing treatment, and HLA-DQB1*05:03 associated with extrapulmonary sarcoidosis.

### 4.1. HLA in sarcoidosis patients compared with the healthy population

This first detailed analysis of the HLA variants in 6 loci (*HLA-A,-B,-C,-DRB1,-DQA1*, and *-DQB1*) could compare so far the highest number of patients and control subjects from the Czech population.

In line with their role in sarcoidosis immunopathogenesis (1, 8, 18), HLA class II loci emerged as the central location of the disease-associated variants: HLA-DQB1*06:02, here defined as a risk variant; was previously reported to be associated mainly with a worse prognosis in sarcoidosis in Swedish and Dutch patients (14, 19) and linked with variants HLA-DRB1*15:01 and HLA-B*07 (15). In our study, HLA-DRB1*15:01 and HLA-B*07:02 were also observed as risk factors for sarcoidosis at the primary significance level. As these two variants were reported in linkage disequilibrium with the HLA-DQB1*06:02 variant (Table 7), it may be speculated that the HLA-DQB1 locus allele is responsible for the observed association.

In this context, we newly characterized variant HLA-DQB1*06:04 as a risk factor for sarcoidosis development, contrary to a previous report from the Netherlands, where there was no difference in the occurrence of this variant in patients compared to the controls (15). The discrepancy could reflect different cohort sizes and distinct ethnic backgrounds of the investigated patients (8).

Variant HLA-DRB1*01:01 was previously reported as protective against sarcoidosis in the population of the United Kingdom, Netherlands, Finland, and Japan, where it was identified as part of the haplotype DQA1*01:01 ∼ DQB1*05:01 ∼ DRB1*01:01; all these variants we report to be in LD and act as protective against sarcoidosis development (17, 20, 21). Except for HLA-DRB1*01:01, which occurred more frequently in our Czech control population than in our patients, the other two protective variants were HLA-DQA1*03:01 and HLA-DQB1*03:02. HLA-DQB1*03:02 was recently reported as protective from sarcoidosis also in Korean population (13). HLA-DQA1*03:01 has so far never been mentioned in connection with sarcoidosis. A hereby observed association can, however, result from LD between HLA-DQA1*03:01 and HLA-DQB1*03:02, and another protective variant–HLA-DRB1*04:01, which we report to protect on the primary level. These three variants are part of one of the most common haplotypes in Caucasian populations (22). Another common haplotype, HLA-DQB1*06:02 ∼ HLA-DQA1*01:02, relates to the risk of sarcoidosis in this study.

### 4.2. HLA and clinical phenotypes of sarcoidosis

We determined variant HLA-DRB1*11:01 as a risk factor for more advanced CXR stages of sarcoidosis. This variant was previously reported to relate to sarcoidosis in general across a spectrum of ethnicities (9), but we are the first to connect this variant with higher CXR stages of the disease, specifically for the Czech population.

Regarding Löfgren’s syndrome, we report six variants (HLA-A*01:01∼HLA-B*08:01∼HLA-C*07:01∼HLA-DRB1*03:01 ∼HLA-DQA1*05:01∼HLA-DQB1*02:01), forming 8.1 haplotype, to be associated with LS. The 8.1 ancestral haplotype is most common in Northern Europe and was associated with risk for autoimmune diseases (23), it was also described as associated with sarcoidosis in populations from Northern Europe (18, 24, 25). The linkage of variants HLA-B*08, HLA-DRB1*03:01, and HLA-DQB1*02:01 with good prognosis of sarcoidosis was observed previously in a Croatian population (26). A favorable disease course was also previously linked to HLA-DQB1*02:01 (19, 27).

Some of the variants of the 8.1 haplotype, HLA-DRB1*03:01 and HLA-DQA1*05:01, were associated with a milder disease course, i.e., initial chest X-ray (CXR 1) or remitting disease. Further, together with HLA-A*01:01, these variants occurred more frequently in patients not requiring corticosteroid treatment. Our observations, therefore, extend findings from previous reports from sarcoidosis association studies across populations that showed the link between HLA-DRB1*03:01 and sarcoidosis patients, especially in the group with a better prognosis (11, 27, 28). We can also speculate about the effect of HLA-DRB1*03:01 homozygosity on better prognosis as four of five homozygotes among our Czech sarcoidosis patients had a remitting disease, a similar relationship was previously observed in HLA-DRB1*03:01 homozygous patients with remission of multiple sclerosis (29). The association of variant HLA-B*08:01 with the milder disease and especially with LS has also been previously reported (27, 30). Concerning our observation of HLA-DQA1*05:01 associating with better prognosis, this variant was reported to be overrepresented in Japanese sarcoidosis patients compared with controls (31), and HLA-A*01:01 was reported with the resolving course of disease in Sweden (27).

Regarding systemic disease, previous reports from Swedish, UK, and Turkish populations associated alleles of the HLA-DRB1 locus (HLA- DRB1*04 -DRB1*15) with extrapulmonary sarcoidosis (16, 32, 33). In our study, extrapulmonary involvement was associated with another class II locus, *HLA-DQB1*: the variant HLA-DQB1*05:03, previously mentioned in connection with sarcoidosis uveitis in UK patients (16), was associated with extrapulmonary disease in our Czech patients. Further, HLA-DQB1*05:03 was associated with persistent disease on the primary level, as well as HLA-DRB1*14:54 and HLA-DQA1*01:04; these three variants occur in LD in the Czech population. In an Indian population, these variants were associated with the risk of sarcoidosis (34).

In context of treatment requirement, there has been scarce information on the relationship between immunogenetic markers and non-requirement for corticosteroid therapy. In a previous study, the presence of the mutant CCR5Delta32 allele carried an increased relative risk of the need for treatment in another cohort of Czech sarcoidosis patients (35). Here we observed that HLA-A*02:01 carriers require treatment two times more often than patients not carrying this variant. As corticosteroid treatment is indicated for patients with significant pulmonary sarcoidosis involvement, who are expected to have a higher risk of future mortality or permanent disability (36), the availability of an additional laboratory marker to be included in the complex decision for treatment initiation may be useful. However, before potential clinical application, this finding must be replicated. On the other hand, no need for treatment was associated with three alleles of the 8.1 haplotype and this observation fits well with the hereby reported link of this haplotype with milder sarcoidosis.

### 4.3. Study strength, limitations, and conclusions

The main strength of this study is the large number of patients characterized in detail and enrolled in a single medical center, allowing comparisons between adequately sized subgroups according to clinical diagnostic standards. We analyzed data from 301 precisely characterized sarcoidosis patients with representative phenotypes. In this context, a low number of patients in CXR stage 4 was the main limitation, the inability to address the allele primarily responsible for an association in cases of linked allele pairs may also be considered limiting. The methodology employed for HLA-typing represents another substantial advantage over previous studies as the precise NGS technique allowed indeterminate characterization at the allele level. Certain limitation of the clinical utility of hereby newly reported results is their validity in a Czech population, unless further replicated elsewhere. On the other hand, the study results could be applied in the meta-analyses of sarcoidosis immunogenetic data.

In conclusion, while the HLA region has long been consistently associated with sarcoidosis, the majority of the reports focused on populations of Northern/Western Europe origin and pointed to class II loci, namely the *HLA-DRB1*, in line with its major role in antigen presentation (1, 8, 18). In this context, our study provides further information obtained from patients of the Slavonic population from Central Europe, and re-emphasizes the possible role of another class II locus—*HLA-DQB1*. Study findings nominate a new susceptibility variant for sarcoidosis, HLA-DQB1*06:04, and extend the previous knowledge on HLA-DQB1*06:02 as a sarcoidosis susceptibility marker to the Czech population. Our study also suggests that the variant HLA-DRB1*11:01, previously reported as a general susceptibility factor, may serve as a marker for the advancing course of the disease. Further, other HLA variants associated with extrapulmonary involvement and the requirement for treatment are reported in our study. Patients with these HLA markers should, therefore, be carefully followed up, screened for extrapulmonary involvement, and considered for treatment.

Finally, a possible contribution of the “autoimmune” 8.1 ancestral haplotype to the better sarcoidosis prognosis, is another important outcome of our study. Association of Löfgren’s syndrome and other milder disease forms with the combination of the following six variants, HLA-A*01:01∼HLA-B*08:01∼HLA-C*07:01∼HLA-DRB1*03:01∼HLA-DQA1*05:01∼ HLA-DQB1*02:01, may be, therefore, considered in the context of the personalized approach to sarcoidosis patients.

## Previous presentations

Part of this work has been presented as an abstract at the 2021 ERS (European Respiratory Society) International Congress, September 5.-8., 2021.

## Data availability statement

The datasets for this article are not publicly available due to concerns regarding participant/patient anonymity and confidentiality. Requests to access the datasets should be directed to the corresponding author.

## Ethics statement

The studies involving human participants were reviewed and approved by the Ethics Committee of the University Hospital and the Faculty of Medicine Palacký University in Olomouc, vote on project NV18-05-00134 dated 27.06.2017. The patients/participants provided their written informed consent to participate in this study.

## Author contributions

MP and MD: conception and design. MD, MP, JP, and MF-V: provision of study materials. KS, MD, MP, KO, MF-V, JP, LK, and AS: collection and assembly of data, manuscript writing, and final approval of the manuscript. KS, MP, KO, MD, and MF-V: data analysis and interpretation. All authors contributed to the article and approved the submitted version.

## Footnotes

1
https://www.hiv.lanl.gov/content/immunology/hla/index.html
