# Supplementary material for: Association between sarcoidosis and HLA polymorphisms in a Czech population from Central Europe: focus on a relationship with clinical outcome and treatment
Source: Front Med (Lausanne). 2023 Apr 21;10:1094843. doi: 10.3389/fmed.2023.1094843 (PMC10160604; doi:10.3389/fmed.2023.1094843)
Supplement: Supplementary file 1 [file Table_1.pdf]

Supplementary Material (Sikorova et al.)

Supplementary Table 1: The distinguished and disease-associated variants in the seven investigated HLA loci (4-digit resolution level). The table shows the number of variants that we were able to distinguish by NGS HLA genotyping and the number of sarcoidosis-associated variants in the investigated HLA loci observed in our Czech patients. Notes: "Genotyping rate" denotes the proportion of correctly assigned HLA genotypes from all sequenced samples; Significant association: on "primary level": $p<0.05$, before the correction for multiple comparisons (*). *HLA-DQB1* locus was typed in all control samples and in 205 out of 301 patient samples (*#*). NA (in the column "Associations") - Not Analysed (HLA-DPB1 locus was excluded from analyses due to a suboptimal typing rate in patients).

	**Patients**		**Controls**		**Overall**		**Associations**	
	**Determined variants**	**Genotyping rate**	**Determined variants**	**Genotyping rate**	**Determined variants**	**Genotyping rate**	**Primary level**	**After correction***
HLA-A	28	100%	24	96%	31	98%	1	0
HLA-B	47	100%	45	98%	55	97%	2	0
HLA-C	30	100%	23	99%	30	100%	0	0
HLA-DRB1	43	100%	37	98%	49	99%	7	1
HLA-DQA1	17	99%	16	95%	18	97%	4	1
HLA-DQB1#	28	95%	17	99%	29	97%	4	3
HLA-DPB1	38	84%	22	99%	40	91%	NA	NA

Supplementary Table 2: Data on the occurrence of the alleles at the *HLA-A, -B, -C, -DRB1, -DQA1* and *-DQB1* loci, which we were able to distinguish by NGS HLA genotyping in samples from Czech patients with sarcoidosis (n=301). *HLA-DQB1* locus was typed in 205 out of 301 patient samples. Allele count – absolute number (A) of a given allele detected in the patient group, Allele frequency (*f*) – relative occurrence of a given allele (*f* = A / N), N-count of all alleles detected in a locus.

**Allele**	**Allele count**	**Allele Frequency**
HLA-A*01:01	108	0.179
HLA-A*01:03	1	0.002
HLA-A*02:01	148	0.246
HLA-A*02:05	5	0.008
HLA-A*02:17	2	0.003
HLA-A*02:30	1	0.002
HLA-A*03:01	84	0.14
HLA-A*03:02	3	0.005
HLA-A*11:01	40	0.066
HLA-A*23:01	20	0.033
HLA-A*24:02	63	0.105
HLA-A*24:03	1	0.002
HLA-A*25:01	33	0.055
HLA-A*26:01	16	0.027
HLA-A*29:01	1	0.002
HLA-A*29:02	6	0.01
HLA-A*30:01	10	0.017
HLA-A*30:02	1	0.002
HLA-A*30:04	1	0.002
HLA-A*31:01	11	0.018
HLA-A*31:48	1	0.002
HLA-A*32:01	17	0.028
HLA-A*33:01	4	0.007
HLA-A*33:03	3	0.005
HLA-A*34:02	1	0.002
HLA-A*66:01	3	0.005
HLA-A*68:01	17	0.028
HLA-A*68:02	1	0.002
HLA-B*07:02	89	0.149
HLA-B*07:04	2	0.003
HLA-B*07:05	1	0.002
HLA-B*08:01	89	0.149
HLA-B*13:02	27	0.045
HLA-B*14:01	2	0.003
HLA-B*14:02	8	0.013
HLA-B*15:01	33	0.055
HLA-B*15:07	1	0.002

HLA-B*15:09	1	0.002
HLA-B*15:17	6	0.01
HLA-B*18:01	48	0.08
HLA-B*18:03	1	0.002
HLA-B*27:02	6	0.01
HLA-B*27:05	18	0.03
HLA-B*35:01	21	0.035
HLA-B*35:02	10	0.017
HLA-B*35:03	19	0.032
HLA-B*35:08	2	0.003
HLA-B*35:16	1	0.002
HLA-B*37:01	5	0.008
HLA-B*38:01	27	0.045
HLA-B*39:01	7	0.012
HLA-B*40:01	24	0.04
HLA-B*40:02	9	0.015
HLA-B*40:06	1	0.002
HLA-B*41:01	1	0.002
HLA-B*41:02	3	0.005
HLA-B*44:02	28	0.047
HLA-B*44:03	19	0.032
HLA-B*44:05	4	0.007
HLA-B*44:27	3	0.005
HLA-B*45:01	1	0.002
HLA-B*47:01	2	0.003
HLA-B*48:01	1	0.002
HLA-B*49:01	9	0.015
HLA-B*50:01	4	0.007
HLA-B*51:01	23	0.038
HLA-B*51:08	1	0.002
HLA-B*52:01	7	0.012
HLA-B*53:01	2	0.003
HLA-B*55:01	6	0.01
HLA-B*56:01	7	0.012
HLA-B*56:04	1	0.002
HLA-B*57:01	15	0.025
HLA-B*58:01	3	0.005
HLA-B*78:02	1	0.002
HLA-C*01:02	20	0.033
HLA-C*02:02	32	0.053
HLA-C*02:13	1	0.002
HLA-C*02:43	1	0.002
HLA-C*03:02	1	0.002
HLA-C*03:03	28	0.047
HLA-C*03:04	31	0.052

HLA-C*04:01	64	0.106
HLA-C*05:01	28	0.047
HLA-C*06:02	55	0.091
HLA-C*07:01	116	0.193
HLA-C*07:02	89	0.148
HLA-C*07:04	9	0.015
HLA-C*07:06	1	0.002
HLA-C*07:16	1	0.002
HLA-C*07:18	2	0.003
HLA-C*07:31	2	0.003
HLA-C*08:01	1	0.002
HLA-C*08:02	10	0.017
HLA-C*12:02	7	0.012
HLA-C*12:03	75	0.125
HLA-C*14:02	7	0.012
HLA-C*15:02	10	0.017
HLA-C*15:04	1	0.002
HLA-C*15:05	1	0.002
HLA-C*16:01	3	0.005
HLA-C*16:02	1	0.002
HLA-C*16:04	1	0.002
HLA-C*17:01	1	0.002
HLA-C*17:03	3	0.005
HLA-DRB1*01:01	16	0.027
HLA-DRB1*01:02	5	0.008
HLA-DRB1*01:03	1	0.002
HLA-DRB1*03:01	75	0.125
HLA-DRB1*03:04	1	0.002
HLA-DRB1*03:05	1	0.002
HLA-DRB1*03:07	1	0.002
HLA-DRB1*04:01	22	0.037
HLA-DRB1*04:02	3	0.005
HLA-DRB1*04:03	4	0.007
HLA-DRB1*04:04	5	0.008
HLA-DRB1*04:05	2	0.003
HLA-DRB1*04:07	2	0.003
HLA-DRB1*04:08	3	0.005
HLA-DRB1*07:01	56	0.093
HLA-DRB1*08:01	10	0.017
HLA-DRB1*08:03	1	0.002
HLA-DRB1*08:04	1	0.002
HLA-DRB1*09:01	6	0.01
HLA-DRB1*10:01	5	0.008
HLA-DRB1*11:01	66	0.11

HLA-DRB1*11:03	3	0.005
HLA-DRB1*11:04	33	0.055
HLA-DRB1*11:11	1	0.002
HLA-DRB1*11:27	1	0.002
HLA-DRB1*11:30	1	0.002
HLA-DRB1*12:01	12	0.02
HLA-DRB1*12:03	1	0.002
HLA-DRB1*12:10	1	0.002
HLA-DRB1*13:01	50	0.083
HLA-DRB1*13:02	29	0.048
HLA-DRB1*13:03	13	0.022
HLA-DRB1*13:05	1	0.002
HLA-DRB1*14:01	1	0.002
HLA-DRB1*14:04	5	0.008
HLA-DRB1*14:15	1	0.002
HLA-DRB1*14:54	20	0.033
HLA-DRB1*15:01	113	0.188
HLA-DRB1*15:02	6	0.01
HLA-DRB1*15:11	1	0.002
HLA-DRB1*15:14	1	0.002
HLA-DRB1*16:01	17	0.028
HLA-DRB1*16:02	3	0.005
HLA-DQA1*01:01	21	0.035
HLA-DQA1*01:02	162	0.272
HLA-DQA1*01:03	57	0.096
HLA-DQA1*01:04	25	0.042
HLA-DQA1*01:05	5	0.008
HLA-DQA1*02:01	56	0.094
HLA-DQA1*03:01	25	0.042
HLA-DQA1*03:02	5	0.008
HLA-DQA1*03:03	19	0.032
HLA-DQA1*04:01	11	0.019
HLA-DQA1*04:02	1	0.002
HLA-DQA1*05:01	71	0.119
HLA-DQA1*05:03	1	0.002
HLA-DQA1*05:05	128	0.215
HLA-DQA1*05:09	5	0.008
HLA-DQA1*05:10	2	0.003
HLA-DQA1*06:01	2	0.003
HLA-DQB1*02:01	40	0.103
HLA-DQB1*02:02	35	0.09
HLA-DQB1*02:16	1	0.003
HLA-DQB1*02:47	1	0.003
HLA-DQB1*03:01	69	0.178
HLA-DQB1*03:02	8	0.021

HLA-DQB1*03:03	12	0.031
HLA-DQB1*03:04	2	0.005
HLA-DQB1*03:05	1	0.003
HLA-DQB1*03:12	1	0.003
HLA-DQB1*03:27	1	0.003
HLA-DQB1*03:28	1	0.003
HLA-DQB1*04:02	5	0.013
HLA-DQB1*05:01	20	0.052
HLA-DQB1*05:02	14	0.036
HLA-DQB1*05:03	22	0.057
HLA-DQB1*05:16	1	0.003
HLA-DQB1*05:18	1	0.003
HLA-DQB1*06:01	4	0.01
HLA-DQB1*06:02	81	0.209
HLA-DQB1*06:03	38	0.098
HLA-DQB1*06:04	21	0.054
HLA-DQB1*06:07	1	0.003
HLA-DQB1*06:09	4	0.01
HLA-DQB1*06:12	1	0.003
HLA-DQB1*06:13	1	0.003
HLA-DQB1*06:39	1	0.003
HLA-DQB1*06:84	1	0.003

Note: In the patient's population, the count of alleles determined on four digits resolution was for the *HLA-A* locus, N = 602; *HLA-B* locus, N = 598; *HLA-C* locus, N = 602; *HLA-DRB1* locus, N = 600; *HLA-DQA1* locus, N = 596; and *HLA-DQB1* locus, N = 388.

Supplementary Table 3: Data on the occurrence of the alleles at the *HLA-A, -B, -C, -DRB1*, *-DQA1* and *-DQB1* loci, which we were able to distinguish by NGS HLA genotyping in samples from Czech control population (n=309). Allele count – absolute number (A) of a given allele detected in the patient group, Allele frequency (*f*) – relative occurrence of a given allele (*f* = A / N), N-count of all alleles detected in a locus.

Allele	Allele count	Allele Frequency
HLA-A*01:01	91	0.153
HLA-A*01:06	1	0.002
HLA-A*02:01	158	0.266
HLA-A*02:05	6	0.01
HLA-A*02:11	1	0.002
HLA-A*02:17	2	0.003
HLA-A*03:01	76	0.128
HLA-A*11:01	30	0.051
HLA-A*23:01	16	0.027
HLA-A*24:02	58	0.098
HLA-A*25:01	27	0.046
HLA-A*26:01	30	0.051
HLA-A*29:01	2	0.003
HLA-A*29:02	10	0.017
HLA-A*30:01	10	0.017
HLA-A*30:04	2	0.003
HLA-A*31:01	15	0.025
HLA-A*32:01	20	0.034
HLA-A*33:01	5	0.008
HLA-A*33:03	2	0.003
HLA-A*66:01	6	0.01
HLA-A*68:01	20	0.034
HLA-A*68:02	2	0.003
HLA-A*69:01	3	0.005
HLA-B*07:02	61	0.101
HLA-B*07:04	1	0.002
HLA-B*07:05	1	0.002
HLA-B*08:01	63	0.104
HLA-B*08:09	1	0.002
HLA-B*13:02	35	0.058
HLA-B*14:01	1	0.002
HLA-B*14:02	10	0.017
HLA-B*15:01	36	0.06
HLA-B*15:17	3	0.005
HLA-B*15:18	1	0.002
HLA-B*15:22	1	0.002
HLA-B*18:01	49	0.081

HLA-B*27:02	8	0.013
HLA-B*27:05	25	0.041
HLA-B*27:14	1	0.002
HLA-B*35:01	30	0.05
HLA-B*35:02	9	0.015
HLA-B*35:03	15	0.025
HLA-B*35:08	2	0.003
HLA-B*37:01	5	0.008
HLA-B*38:01	21	0.035
HLA-B*39:01	10	0.017
HLA-B*39:06	3	0.005
HLA-B*40:01	17	0.028
HLA-B*40:02	8	0.013
HLA-B*40:06	4	0.007
HLA-B*41:01	3	0.005
HLA-B*41:02	6	0.01
HLA-B*41:18	1	0.002
HLA-B*44:02	39	0.065
HLA-B*44:03	20	0.033
HLA-B*44:05	6	0.01
HLA-B*44:27	9	0.015
HLA-B*45:01	3	0.005
HLA-B*49:01	5	0.008
HLA-B*50:01	9	0.015
HLA-B*51:01	35	0.058
HLA-B*52:01	12	0.02
HLA-B*53:05	1	0.002
HLA-B*55:01	5	0.008
HLA-B*56:01	6	0.01
HLA-B*57:01	19	0.031
HLA-B*58:01	3	0.005
HLA-B*78:01	1	0.002
HLA-C*01:02	29	0.047
HLA-C*02:02	40	0.065
HLA-C*03:02	2	0.003
HLA-C*03:03	25	0.041
HLA-C*03:04	34	0.055
HLA-C*04:01	74	0.121
HLA-C*05:01	38	0.062
HLA-C*06:02	66	0.108
HLA-C*07:01	92	0.15
HLA-C*07:02	71	0.116

HLA-C*07:04	15	0.024
HLA-C*07:18	2	0.003
HLA-C*08:02	10	0.016
HLA-C*12:02	13	0.021
HLA-C*12:03	58	0.095
HLA-C*14:02	6	0.01
HLA-C*15:02	16	0.026
HLA-C*15:04	2	0.003
HLA-C*15:05	1	0.002
HLA-C*16:01	6	0.01
HLA-C*16:02	3	0.005
HLA-C*17:01	3	0.005
HLA-C*17:03	7	0.011
HLA-DRB1*01:01	41	0.068
HLA-DRB1*01:02	5	0.008
HLA-DRB1*03:01	67	0.11
HLA-DRB1*03:17	1	0.002
HLA-DRB1*04:01	38	0.063
HLA-DRB1*04:02	11	0.018
HLA-DRB1*04:03	4	0.007
HLA-DRB1*04:04	10	0.016
HLA-DRB1*04:05	2	0.003
HLA-DRB1*04:07	3	0.005
HLA-DRB1*04:08	3	0.005
HLA-DRB1*07:01	90	0.148
HLA-DRB1*08:01	14	0.023
HLA-DRB1*08:03	2	0.003
HLA-DRB1*08:04	1	0.002
HLA-DRB1*09:01	7	0.012
HLA-DRB1*10:01	6	0.01
HLA-DRB1*11:01	43	0.071
HLA-DRB1*11:02	3	0.005
HLA-DRB1*11:03	10	0.016
HLA-DRB1*11:04	31	0.051
HLA-DRB1*11:15	1	0.002
HLA-DRB1*11:19	1	0.002
HLA-DRB1*11:28	1	0.002
HLA-DRB1*12:01	8	0.013
HLA-DRB1*13:01	42	0.069
HLA-DRB1*13:02	12	0.02
HLA-DRB1*13:03	14	0.023
HLA-DRB1*13:05	2	0.003
HLA-DRB1*14:01	2	0.003

HLA-DRB1*14:04	5	0.008
HLA-DRB1*14:54	9	0.015
HLA-DRB1*15:01	80	0.132
HLA-DRB1*15:02	13	0.021
HLA-DRB1*15:07	1	0.002
HLA-DRB1*16:01	22	0.036
HLA-DRB1*16:02	2	0.003
HLA-DQA1*01:01	43	0.073
HLA-DQA1*01:02	118	0.201
HLA-DQA1*01:03	51	0.087
HLA-DQA1*01:04	17	0.029
HLA-DQA1*01:05	5	0.009
HLA-DQA1*01:10	1	0.002
HLA-DQA1*02:01	87	0.148
HLA-DQA1*03:01	51	0.087
HLA-DQA1*03:02	5	0.009
HLA-DQA1*03:03	19	0.032
HLA-DQA1*04:01	12	0.02
HLA-DQA1*04:02	1	0.002
HLA-DQA1*05:01	62	0.106
HLA-DQA1*05:03	1	0.002
HLA-DQA1*05:05	111	0.189
HLA-DQA1*06:01	2	0.003
HLA-DQB1*02:01	67	0.109
HLA-DQB1*02:02	65	0.106
HLA-DQB1*03:01	130	0.212
HLA-DQB1*03:02	59	0.096
HLA-DQB1*03:03	29	0.047
HLA-DQB1*03:04	4	0.007
HLA-DQB1*03:19	3	0.005
HLA-DQB1*04:02	14	0.023
HLA-DQB1*05:01	54	0.088
HLA-DQB1*05:02	30	0.049
HLA-DQB1*05:03	19	0.031
HLA-DQB1*06:01	10	0.016
HLA-DQB1*06:02	73	0.119
HLA-DQB1*06:03	45	0.073
HLA-DQB1*06:04	8	0.013
HLA-DQB1*06:09	3	0.005
HLA-DQB1*06:39	1	0.002

Note: In the control population, the count of alleles determined on four digits resolution was for the *HLA-A* locus, N = 594; *HLA-B* locus, N = 604; *HLA-C* locus, N = 612; *HLA-DRB1* locus, N = 608; *HLA-DQA1* locus, N = 586; and *HLA-DQB1* locus, N = 614.

Supplementary Table 4: Comparison of the HLA variant carriage rate between the patients with Löfgren's syndrome, LS (n = 55) compared with the patients with non-LS (n = 244). OR – odds ratio, CI-confidence interval, p_{corr} – *p*-value after the correction for multiple comparisons (significant values highlighted in bold), NA - not analysed. *HLA-DQB1* locus was typed in 31 LS patients and 173 nonLS patients (*#*).

HLA	LS n=55#	nonLS n=244#	OR (95% CI)	*p-value*	p_{corr}
**HLA-B*08:01**	**0.564**	**0.225**	**4.439(2.408-8.183)**	**1.99*10^{-06}**	**9.34*10^{-05}**
HLA-B*41:02	0.055	0	NA	0.006	0.245
HLA-C*04:01	0.073	0.234	0.257(0.089-0.743)	0.005	0.157
**HLA-C*07:01**	**0.582**	**0.283**	**3.529(1.929-6.455)**	**5.71*10^{-05}**	**0.002**
HLA-C*07:18	0.036	0	NA	0.033	0.65
HLA-C*17:03	0.055	0	NA	0.006	0.169
**HLA-DRB*03:01**	**0.582**	**0.156**	**7.506(3.966-14.205)**	**4.48*10^{-10}**	**1.93*10^{-08}**
HLA-DRB*11:01	0.091	0.243	0.312(0.119-0.819)	0.011	0.383
**HLA-DQA*01:02**	**0.2**	**0.502**	**0.248(0.146-0.422)**	**5.45*10^{-08}**	**9.26*10^{-07}**
**HLA-DQA*01:03**	**0.064**	**0.203**	**0.266(0.116-0.609)**	**0.001**	**0.014**
**HLA-DQA*01:04**	**0.009**	**0.095**	**0.087(0.012-0.652)**	**0.002**	**0.037**
**HLA-DQA*02:01**	**0.073**	**0.195**	**0.324(0.147-0.711)**	**0.003**	**0.044**
**HLA-DQA*05:01**	**0.3**	**0.145**	**2.522(1.466-4.341)**	**0.001**	**0.02**
**HLA-DQA*05:05**	**0.173**	**0.402**	**0.31(0.178-0.541)**	**1.63*10^{-05}**	**2.77*10^{-04}**
HLA-DQB*03:01	0.15	0.301	0.411(0.188-0.899)	0.025	0.512
HLA-DQB*03:03	0	0.074	NA	0.039	0.674
HLA-DQB*05:03	0	0.098	NA	0.007	0.189
HLA-DQB*06:02	0.167	0.344	0.382(0.18-0.811)	0.013	0.299

Supplementary Table 5: Comparison of the HLA variant carriage rate between the patients with chest X-ray (CXR) stage 1 (n = 95) compared with the patients with stages 2-4 (n = 206); only the alleles associated at least on the primary level are shown. For the legend see table Supplementary Table 2. *HLA-DQB1* locus was typed in 51 stage 1 patients and 154 stage 2-4 patients (*#)*.

**HLA**	**CXR st. 1 n=95#**	**CXR st. 2-4 n=206#**	**OR (95% CI)**	***p-value***	p_{corr}
HLA-A*01:01	0.432	0.27	2.057(1.235-3.427)	0.008	0.192
**HLA-B*08:01**	**0.421**	**0.225**	**2.498(1.48-4.215)**	**0.001**	**0.042**
HLA-B*35:01	0.021	0.083	0.237(0.054-1.046)	0.043	0.871
HLA-B*38:01	0.147	0.064	2.539(1.143-5.643)	0.029	0.743
HLA-B*41:02	0.032	0	NA	0.031	0.777
HLA-B*44:03	0.021	0.083	0.237(0.054-1.046)	0.043	0.871
**HLA-C*07:01**	**0.484**	**0.27**	**2.543(1.531-4.225)**	$\mathbf{3.70*10^{-04}}$	**0.011**
HLA-C*17:03	0.032	0	NA	0.031	0.628
**HLA-DRB1*03:01**	**0.411**	**0.148**	**4.016(2.286-7.054)**	$\mathbf{1.46*10^{-06}}$	$\mathbf{6.29*10^{-05}}$
**HLA-DRB1*11:01**	**0.074**	**0.276**	**0.209(0.091-0.478)**	$\mathbf{3.20*10^{-05}}$	**0.001**
**HLA-DQA1*05:01**	**0.419**	**0.138**	**4.514(2.544-8.009)**	$\mathbf{2.13*10^{-07}}$	$\mathbf{3.63*10^{-06}}$
HLA-DQA1*05:05	0.269	0.443	0.462(0.27-0.789)	0.005	0.077
HLA-DQB1*02:01	0.319	0.11	3.809(1.705-8.507)	0.002	0.061

Supplementary Table 6: Comparison of the HLA variant carriage rate between the patients with disease remission (n = 142) compared with the patients with persistent disease course (n = 134); only the alleles associated at least on the primary level are shown. For the legend see table Supplementary Table 2. *HLA-DQB1* locus was typed in 80 patients in remission and 100 patients with persistent disease (*#)*.

**HLA**	**Remission n=142#**	**Persistence n=134#**	**OR (95% CI)**	***p-value***	p_{corr}
HLA-A*01:01	0.415	0.254	2.091(1.252-3.491)	0.005	0.133
HLA-B*07:02	0.218	0.328	0.571(0.334-0.977)	0.043	0.875
HLA-B*08:01	0.359	0.209	2.122(1.237-3.639)	0.008	0.299
HLA-B*35:03	0.092	0.022	4.401(1.225-15.807)	0.019	0.585
HLA-B*38:01	0.134	0.06	2.433(1.027-5.764)	0.044	0.877
HLA-B*44:03	0.028	0.104	0.248(0.08-0.775)	0.013	0.47
**HLA-DRB1*03:01**	**0.352**	**0.113**	**4.275(2.259-8.093)**	**$3.83*10^{-06}$**	**$1.65*10^{-04}$**
HLA-DRB1*07:01	0.12	0.241	0.429(0.225-0.817)	0.011	0.387
HLA-DRB1*09:01	0	0.038	NA	0.025	0.67
HLA-DRB1*11:01	0.141	0.286	0.41(0.224-0.75)	0.005	0.183
HLA-DRB1*14:54	0.028	0.113	0.228(0.074-0.706)	0.008	0.281
HLA-DQA1*01:04	0.036	0.128	0.253(0.09-0.706)	0.007	0.108
HLA-DQA1*02:01	0.121	0.241	0.436(0.229-0.831)	0.012	0.181
**HLA-DQA1*05:01**	**0.35**	**0.113**	**4.236(2.234-8.031)**	**$3.55*10^{-06}$**	**$6.03*10^{-05}$**
HLA-DQB1*05:03	0.027	0.117	0.207(0.044-0.963)	0.04	0.68
HLA-DQB1*06:09	0.053	0	NA	0.037	0.653

Supplementary Table 7: Comparison of the HLA variant carriage rate between the patients with treatment (n = 194) compared with the patients without treatment (n = 105); only the alleles associated at least on the primary level are shown. For the legend see table Supplementary Table 2. *HLA-DQB1* locus was typed in 146 patients with corticosteroid treatment and 58 patients without treatment (*#*).

HLA	treatment yes, n=194*#*	treatment no, n=105*#*	OR (95% CI)	*p-value*	p_{corr}
**HLA-A*01:01**	**0.232**	**0.495**	**0.308(0.185-0.511)**	**$5.18*10^{-06}$**	**$1.45*10^{-04}$**
**HLA-A*02:01**	**0.49**	**0.295**	**2.291(1.382-3.796)**	**0.001**	**0.038**
HLA-A*03:01	0.304	0.181	1.978(1.104-3.545)	0.027	0.533
HLA-A*33:03	0	0.029	NA	0.043	0.704
HLA-B*08:01	0.237	0.381	0.505(0.302-0.845)	0.011	0.403
HLA-B*35:01	0.036	0.114	0.29(0.111-0.761)	0.012	0.432
HLA-B*58:01	0	0.029	NA	0.043	0.87
HLA-C*04:01	0.16	0.286	0.475(0.268-0.842)	0.016	0.386
HLA-C*07:01	0.294	0.419	0.577(0.351-0.947)	0.03	0.616
**HLA-DRB1*03:01**	**0.171**	**0.352**	**0.379(0.219-0.656)**	**0.001**	**0.024**
HLA-DRB1*11:01	0.259	0.133	2.273(1.188-4.346)	0.012	0.407
**HLA-DQA1*05:01**	**0.165**	**0.353**	**0.362(0.208-0.631)**	**$4.30*10^{-04}$**	**0.007**
HLA-DQA1*05:05	0.448	0.284	2.047(1.223-3.425)	0.006	0.098
HLA-DQB1*02:01	0.122	0.278	0.362(0.166-0.792)	0.016	0.368
HLA-DQB1*05:03	0.108	0.019	6.411(0.826-49.786)	0.045	0.722
